# Mid-term clinical results of chronic cavitary long bone osteomyelitis treatment using S53P4 bioactive glass: a multi-center study

**DOI:** 10.5194/jbji-6-413-2021

**Published:** 2021-11-12

**Authors:** Tom A. G. Van Vugt, Jeffrey Heidotting, Jacobus J. Arts, Joris J. W. Ploegmakers, Paul C. Jutte, Jan A. P. Geurts

**Affiliations:** 1 Department of Orthopedic Surgery, CAPHRI Care and Public Health Research Institute, Maastricht University Medical Centre (MUMC+), Maastricht, the Netherlands; 2 Department of Orthopedic Surgery, University Medical Center Groningen (UMCG), Groningen, the Netherlands; 3 Department of Biomedical Engineering (research group Orthopaedic Biomechanics), Eindhoven University of Technology, Eindhoven, the Netherlands

## Abstract

**Introduction**: Chronic osteomyelitis is a challenging condition in the orthopedic practice and traditionally treated using local and systemic antibiotics in a two-stage surgical procedure. With the introduction of the antimicrobial biomaterial S53P4 bioactive glass (Bonalive^®^), chronic osteomyelitis can be treated in a one-stage procedure. This study evaluated the mid-term clinical results of patients treated with S53P4 bioactive glass for long bone chronic osteomyelitis. **Methods**: In this prospective multi-center study, patients from two different university medical centers in the Netherlands were included. One-stage treatment consisted of debridement surgery, implantation of S53P4 bioactive glass, and treatment with culture-based systemic antibiotics. If required, wound closure by a plastic surgeon was performed. The primary outcome was the eradication of infection, and a secondary statistical analysis was performed on probable risk factors for treatment failure. **Results**: In total, 78 patients with chronic cavitary long bone osteomyelitis were included. Follow-up was at least 12 months (mean 46; standard deviation, SD, 20), and 69 patients were treated in a one-stage procedure. Overall infection eradication was 85 %, and 1-year infection-free survival was 89 %. Primary closure versus local/muscular flap coverage is the only risk factor for treatment failure. **Conclusion**: With 85 % eradication of infection, S53P4 bioactive glass is an effective biomaterial in the treatment of chronic osteomyelitis in a one-stage procedure. A major risk factor for treatment failure is the necessity for local/free muscle flap coverage. These results confirm earlier published data, and together with the fundamentally different antimicrobial pathways without antibiotic resistance, S53P4 bioactive glass is a recommendable biomaterial for chronic osteomyelitis treatment and might be beneficial over other biomaterials.

## 1 Introduction

Chronic osteomyelitis is a bacterial infection of the bone and bone marrow and is one of the biggest clinical challenges in current orthopedic practice. It is predominantly post-traumatic and is often seen after orthopedic (open) fracture surgery but can also be caused by a hematogenous spread or direct postoperative colonization (Lazzarini et al., 2004; Lew and Waldvogel, 2004). Treatment of these infections is difficult because the infected bone and the surrounding tissues are often devitalized (i.e., dead bone sequesters) and poorly vascularized due to the (mostly) long-term present infection. In combination with risk factors as smoking, peripheral vascular disease, diabetes or malnutrition, patients often need multiple surgical procedures where the risks of reinfection (up to 20 %–30 %) remain relatively high (Garcia Del Pozo et al., 2018; Tice et al., 2003).

**Figure 1:**
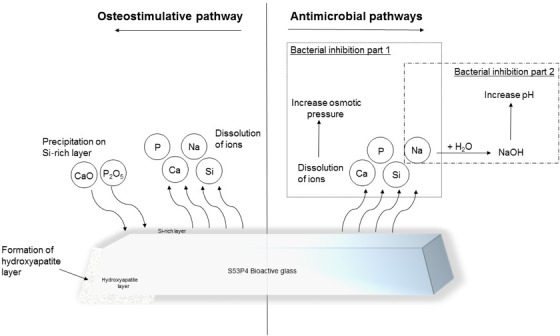
Illustration of the osteostimulative and antimicrobial pathways of S53P4 bioactive glass (Bonalive^®^).

Treatment of chronic osteomyelitis is based on extensive surgical debridement, culture based local and systemic antibacterial/antibiotic therapy, proper dead space management and good soft tissue coverage (Walter et al., 2012). Traditionally this was performed in a two-stage procedure with temporary placement of antibiotic-loaded polymethylmethacrylate (PMMA) beads or spacers. During the second stage, these temporary fillers were removed, and the bone defect is filled with autograft, allograft, or a bone graft substitute (Blaha et al., 1993; Shih et al., 2005). Disadvantages of this treatment algorithm are the need for a second surgery, possible inoculation/biofilm formation of the antibiotic-loaded PMMA implants, and the possibility of thermal damage to the surrounding tissues. In the past few years, multiple antibacterial biodegradable bone graft substitutes have been developed and tested to address these disadvantages (Ferguson et al., 2017; Geurts et al., 2020). Examples of these different biomaterials are antibiotic-loaded calcium sulfates, antibiotic-loaded calcium phosphates, and antibiotic-loaded collagen fleeces/sponges (van Vugt et al., 2016, 2018).

S53P4 bioactive glass (BAG) is a biomaterial with antimicrobial properties that can be used as a bone graft substitute. This S53P4 BAG has been used for the past few years within the fields of oncological, spinal, and traumatic orthopedic surgery but is also used within the field of craniomaxillofacial and otorhinolaryngological surgery (van Gestel et al., 2015). S53P4 BAG is suitable as a bone defect filler after debridement surgery because of its antimicrobial and osteoconductive properties (Fig. 1). After implantation of S53P4 BAG, the ion release causes the formation of a hydroxyapatite layer resulting in osseointegration. After osseointegration, osteogenic cells are activated, resulting in further bone formation, and this activation might be associated with angiogenesis. On the other hand, the immediate ion release after implantation is the basis of the antimicrobial mechanism of S53P4 BAG. The ion release causes a vast increase in local pH and osmotic pressure, leading to bacterial growth inhibition and the destruction of bacteria (Drago et al., 2015; Zhang et al., 2010). Thus, different studies showed that there is no inoculation of S53P4 BAG or biofilm formation on the surface (Coraca-Huber et al., 2014; Bortolin et al., 2016). The antibacterial mechanisms of S53P4 BAG, therefore, are fundamentally different from the regularly known antibacterial mechanisms of the antibiotic-loaded bone graft substitutes.

Several in vitro studies showed that S53P4 BAG can kill a multitude of both gram-positive and gram-negative strains of planktonic bacteria and even bacteria living in biofilms (Lepparanta et al., 2008; Munukka et al., 2008). In addition, there was no sign of antimicrobial resistance against S53P4 BAG in these studies. This is a major benefit over antibiotic-loaded bone graft substitutes and the global increasing problem of antimicrobial resistance (AMR) of bacterial strains (Bortolin et al., 2016; Drago et al., 2014).

This study aims to further substantiate the clinical efficacy regarding treatment of long bone chronic osteomyelitis using S53P4 bioactive glass in a one-stage treatment. In addition to the published literature, secondary analysis on possible risk factors for the treatment failure of infection eradication or treatment related complications was performed.

## 2 Methods

This prospective multi-center cohort study was performed between September 2011 and June 2020. During this period, patients with a confirmed chronic (cavitary) osteomyelitis of the long bones were included from two different university medical centers in the Netherlands, i.e., Maastricht University Medical Centre (MUMC + ) and University Medical Centre Groningen (UMCG). The majority of all patients were treated in a one-stage procedure, with extensive surgical debridement, followed by implantation of S53P4 bioactive glass (Bonalive^®^, Bonalive Biomaterials Ltd, Turku, Finland). Both medical centers had a comparable diagnostic process, treatment algorithm, and follow-up schedule. This study was approved by the Medical Ethical Committee of MUMC + (METC 174084; Maastricht University, 26 May 2017), and all patients signed informed consent.

The primary endpoint of this study was eradication of infection based on a combination of the absence of clinical, radiographic, and/or laboratory parameters of chronic osteomyelitis. Secondary endpoints were the identification of possible parameters related to treatment failure or complications regarding this treatment algorithm.

### 2.1 Patients

Inclusion criteria for enrollment in this study were patients with a clinical, radiological, and/or laboratory test that confirmed the chronic osteomyelitis of a long bone, calcaneal, or pubic bone. The treatment had to require debridement surgery combined with the filling of a bony defect and systemic antimicrobial therapy. Exclusion criteria were chronic osteomyelitis related to diabetic ulcer disease, infected non-unions, or patients who were unable to undergo surgery or long-term antibiotic treatment.

Chronic osteomyelitis was defined as the presence of clinical symptoms for at least 6 weeks (e.g., local signs of infection, fever, and draining sinus tracts), positive preoperative cultures or blood tests (C-reactive protein, leukocyte counts, and erythrocyte sedimentation rate) combined with radiographic imaging (fluorodeoxyglucose (FDG) positron emission tomography (PET)–computed tomography (CT) imaging, magnetic resonance imaging (MRI), or CT-guided bone biopsies). FDG PET-CT imaging was not solely used as a diagnostic imaging modality, but it was also used to evaluate the extend of the infection of the bone and surrounding tissues for preoperative planning (Wang et al., 2011).

Preoperative data were collected based on different patient characteristics, e.g., demographics, medical history, causative mechanism and pathogen, previous surgeries, location, Cierny–Mader classification (Cierny et al., 2003), soft tissue status, and the presence of a sinus tract.

### 2.2 Treatment algorithm

All included patients underwent a one-stage or two-stage treatment algorithm. At the beginning of this study, within the first patients, nine patients with a severe infection were treated in a more conservative way following a two-stage protocol in both centers. After a 1-year clinical follow-up with good preliminary results, this resulted in an adjustment of the treatment protocol to solely one-stage procedures. The one-stage procedure consisted of extensive debridement of infected and necrotic bone and soft tissues, combined with the implantation of S53P4 BAG and systemic-pathogen-specific antibiotics for a total of 6 weeks (2 weeks intravenous and 4 weeks oral). Patients treated with a two-stage surgical procedure underwent extensive debridement of all infected and necrotic bone and soft tissues, combined with implantation of gentamicin-loaded PMMA beads during the first procedure, where, during a second surgery, these beads were removed, and the bone defect was filled with S53P4 BAG. Subsequently, all patients received systemic-pathogen-specific antibiotics comparable to the one-stage treatment group. In both treatment algorithms, soft tissue coverage with a vascularized (free) muscle flap was performed by a plastic surgeon during (the first) surgery, if necessary. After surgery, all patients were treated with systemic culture specific antibiotics for 6 weeks, of which at least 10–14 d was parenteral. During postoperative follow-up, patients underwent laboratory blood tests, X-ray, and (PET-) CT imaging to assess eradication of infection at set moments

### 2.3 Statistics

All data were collected and analyzed using SPSS Statistics v25 (SPSS Inc., Chicago, Illinois, USA). The collected data are considered to be non-parametric, and therefore, a univariate analysis of continuous variables is performed using the Mann–Whitney U test. Associations between different categorical variables was performed using the Fisher's exact test. Data were considered statistically significant if the p value was <0.05 . In the case of missing values, values were replaced by a mean parameter value. In addition, a Kaplan–Meier survival analysis was plotted to visualize the recurrence of chronic osteomyelitis over time, and there were several subgroup analyses performed to analyze the possible influences of variables as one-stage treatment, two-stage treatment, and the necessity for a secondary closure on the effectiveness of S53P4 BAG.

**Figure 2:**
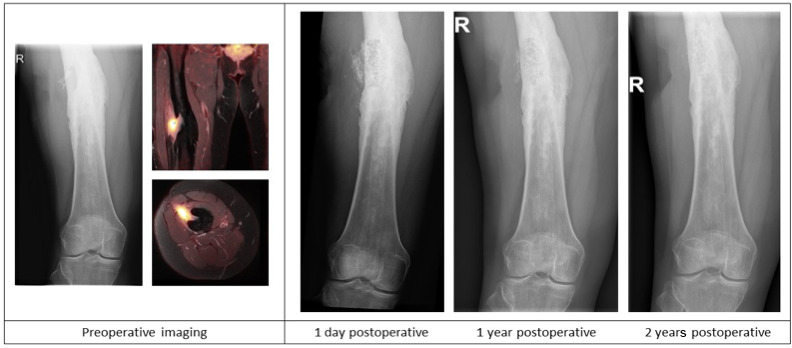
Case description of a 50-year-old female at our outpatient clinic with a painful upper leg and a draining fistula 20 years after an open femoral fracture. Preoperative X-ray and PET-MR imaging showing a chronic intramedullary osteomyelitis of the femur with a fistula and a sequester. Postoperative X-ray imaging after one-stage debridement surgery and implantation of S53P5 bioactive glass show bone defect filling and partial degradation of the S53P5 bioactive glass particles is shown.

## 3 Results

During the inclusion period, a total of 78 patients with chronic osteomyelitis were treated at both hospitals (50 MUMC + ; 28 UMCG). Baseline demographics of the patient cohorts in both hospitals were comparable (Table 1). All patients were followed for at least 1 year, and the average follow-up time was 46 months (SD 20 months). The clear majority of infections was located at the tibia (48 %) and the femur (33 %); other locations were calcaneus (9 %), pubic bones (5 %), distal radius (2.5 %), and iliac crest bone (2.5 %). Chronic osteomyelitis was 69 % of all cases of a post-traumatic origin, where 18 % was due direct post-operative inoculation, 12 % was infected due a hematogenous spread of bacteria, and 1 % remained of unknown origin. Cultured pathogens and a Cierny–Mader classification of all included patients are displayed in Tables 2 and 3. The most common causative pathogen was *staphylococcus aureus*, with 36 % of all cases, where 18 % had a polymicrobial intraoperative cultures, and regardless of the collection of multiple peri-operative tissue samples, 19 % of all cultures were negative. Of all 78 treated patients, 69 patients received a one-stage treatment and 9 patients were treated in a two-stage fashion. In total, 16 of 78 patients required secondary wound (a rotational or free muscle flap) closure by a plastic surgeon.

**Table 1:** Baseline patient demographics.

	Combined	MUMC	UMCG	p value
Age; year ( SD )	54 ( 18 )	53 ( 17 )	55 ( 19 )	0.511
Gender; M/F	55/23	38/12	17/11	0.198
BMI ( SD )	26 ( 6 )	26 ( 6 )	26 ( 6 )	0.621
Smokers ( % )	27 ( 35 )	16 ( 32 )	11 ( 39 )	0.722
Fistula ( % )	37 ( 47 )	26 ( 52 )	11 ( 39 )	0.347
Total	78	50 ( 64 % )	28 ( 36 % )	

SD – standard deviation.

**Table 2:** Causative pathogens.

	Frequency	%
*S. aureus*	28	35.9
*S. epidermidis*	3	3.8
*Staphylococcus sp.*	5	6.4
*Streptococcus sp.*	5	6.4
Other	8	10.3
Polymicrobial	14	17.9
Culture negative	15	19.2
Total	78	100

**Table 3:** Cierny–Mader classification.

	Frequency	%
Intra-medullary	23	29.5
Superficial	3	3.8
Localized	44	56.4
Generalized	8	10.3
Total	78	100

**Table 4:** Treatment/postoperative results.

	Combined	MUMC	UMCG	p value	One stage	Two stage	p value
One vs. two stage	69/9	44/6	25/3	1.000	–	–	–
Closure (Prim/PCH)	62/16	40/10	22/6	1.000	54/15	8/1	0.676
Inf. free at 1 year	89 %	90 %	88 %	0.836	88 %	100 %	0.689
Inf. free at 2 years	84 %	84 %	84 %	0.961	82 %	100 %	0.340
Inf. free last FU	85 %	84 %	86 %	0.841	83 %	100 %	0.340
Follow-up time; mo ( SD )	46 ( 20 )	46 ( 24 )	46 ( 14 )	0.880	43 ( 19 )	86 ( 21 )	0.002
Inf. free period; mo ( SD )	41 ( 25 )	42 ( 27 )	39 ( 22 )	0.836	37.5 ( 23 )	68 ( 21 )	0.001
Complications	13 %	12 %	12 %	0.740	13 %	11 %	1.000
Time to reinfection; mo ( SD )	8.8 ( 8 )	9.6 ( 9 )	7.0 ( 7 )	0.864	9 ( 8 )	n/a	n/a

n/a – not applicable; SD – standard deviation.

### 3.1 Primary results

**Figure 3:**
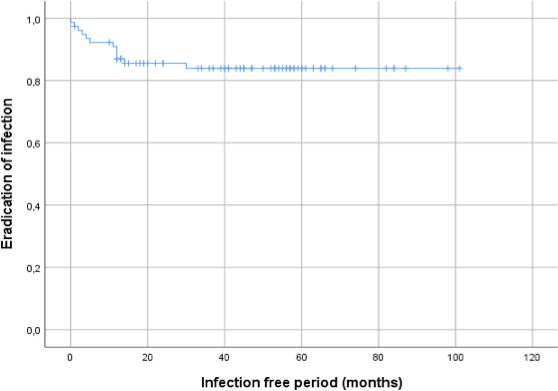
Kaplan–Meier survival curve displaying the infection-free period after initial treatment of all 78 included patients.

A total of 66 patients out of 78 (85 %) showed complete eradication of infection during total clinical follow-up based on clinical presentation, hematological analysis, and additional radiographic and/or nuclear imaging. In total, 68 out of 76 (89 %) of all patients were infection free at the 1-year follow-up, with a loss to follow-up of two patients within the first year. Moreover, 59 out of 70 (84 %) patients were infection free at the 2-year follow-up (Table 4). During follow-up, clinical radiographic images the outpatient clinic showed bone defect healing and partial degradation of S53P4 bioactive glass particles over time (Fig. 2). These radiographic controls did not show any signs of reinfection, although there were 12 patients with a reinfection confirmed by clinical symptoms or PET-CT imaging. The average time until reinfection was 9 months (8.8 months; SD 8 months; Table 4 and Fig. 3). There were four patients who had a major complication (three fractures through the bone window and one failure of osteosynthesis without reinfection), and six patients had a minor complication. All minor complications were persisting wound problems, and five of these six patients had a persisting/early recurrent infection. As mentioned before, a total of two patients (one in each medical center) were lost to follow-up during the first year because they died. One death was related to treatment; this patient died due to massive pulmonary embolisms after a femoral fracture through the bony defect. The other patient died due to multi-organ failure based on pre-existing poor health status, which was not related to infection or treatment. In order to assess and identify the possible influences and confounding effects of two-stage procedures and the necessity for a flap coverage, two subgroup analyses were performed (Tables 4–6). In the first subgroup analysis, we excluded all patients treated in a two-stage fashion. This showed a success rate of 83 % at the end of follow-up, and besides the expected (non-clinically relevant but significant) difference in follow-up time and infection free-period, the analysis did not show any significant changes in our outcomes. In the second subgroup analysis, we excluded all patients who received a soft tissue coverage by a plastic surgeon. This did not change the outcomes.

**Table 5:** Analysis on possible risk factors for recurrence of infection.

	Infection free	Reinfection	p value
Age; year ( SD )	53 ( 19 )	60 ( 13 )	0.210
Gender (M/F)	47/19	8/4	0.741
BMI ( SD )	26 ( 5 )	29 ( 8 )	0.272
Smokers ( % )	33	42	0.743
Cierny–Mader ( 1/2/3/4 )	21/3/35/7	2/0/9/1	0.644
Fistula ( % )	42	75	0.058
Mono-/polybacterial	41/11	7/4	0.435
One vs. two stage	57/9	12/0	0.340
Closure (Prim/PCH)	58/8	4/8	0.001
Complications ${}^{*}$	10 %	25 %	0.178
Total	66	12	

${}^{*}$ There were three × femoral fractures, one × failure of osteosynthesis, and six × persisting wound problems (five resulting in reinfection).

### 3.2 Secondary results

A secondary analysis of all preoperative baseline characteristics did not show a difference between studied variables regarding the risks of recurrence of infection (Table 5). The presence of a preoperative fistula was higher in the reinfection group, but no statistical significance was seen ( p=0.058 ). An analysis of surgery-related variables showed that wound closure, primary closure vs. skin graft, or muscular flap coverage is a major risk factor for the recurrence of infection (12 % vs. 67 % recurrence; p<0.001 ; see Table 5). One-stage treatment vs. two-stage treatment, monobacterial or polybacterial, and culture negative intraoperative cultures are not related to the recurrence of infection in this study. The additional subgroup analysis, as performed for the primary outcomes, did not show any significantly different outcomes in the identification for risk factors for failure and complications (Table 6).

**Table 6:** Subgroup analysis on possible risk factors for recurrence of infection.

	Only patients not needing				Only patients receiving		
	secondary closure				one-stage treatment		
	Free	Reinfection	p value		Free	Reinfection	p value
Age; year ( SD )	51 ( 18 )	50 ( 9 )	0.814		52 ( 19 )	60 ( 13 )	0.234
Gender (M/F)	42/16	4/0	0.565		39/18	8/4	1.000
BMI ( SD )	26 ( 5 )	31 ( 9 )	0.213		25 ( 5 )	29 ( 8 )	0.330
Smokers ( % )	38/20	2/2	0.610		38/19	7/5	0.740
Cierny–Mader ( 1/2/3/4 )	21/3/27/7	0/0/4/0	0.233		17/3/31/6	2/0/9/1	0.567
Primary treatment							
Fistula ( % )	38/20	2/2	0.610		31/26	3/9	0.110
Mono-/polybacterial	36/9	3/1	1.000		34/10	7/4	0.443
Culture negative	45/13	4/0	0.571		44/13	11/1	0.436
Closure (Prim/PCH)	–	–	–		50/7	4/8	0.001
Complications	52/6	3/1	0.389		51/6	9/3	0.183
Total	58	4			57	12	

## 4 Discussion

The results of this study show a total osteomyelitis eradication in 66 of 78 (85 %) included patients after treatment with S53P4 bioactive glass on a combined assessment of clinical presentation, radiographic imaging, and laboratory tests after a mean follow-up of almost 4 years (46 months). During treatment and clinical follow-up, low rates of complications related to S53P4 bioactive glass use were observed. The majority of patients in this cohort study was treated in a one-stage fashion, using S53P4 bioactive glass as a local antimicrobial biomaterial supplemented with systemic antibiotic after surgical debridement. This treatment algorithm is a proven and clinically effective strategy in chronic osteomyelitis treatment.

In addition to these excellent eradication results, this study showed that the necessity for local soft tissue coverage is related to a significant increase in the recurrence of infection. This might be because more severe cases need secondary soft tissue coverage and/or due to local compromised vascular status. Although this is a risk factor for failure, proper soft tissue coverage, with a full thickness skin graft or muscle flap, is necessary since poor postoperative soft tissue status is associated with even higher failure risks (Sanders and Mauffrey, 2013). We would like to point out that an in-depth analysis of the Cierny–Mader classification did not show any correlation between this severity classification and recurrence of infection. This might be due to a relatively high number of patients graded Cierny–Mader class 3 (44 out of 78 patients) in relation to the relatively low number of reinfections within this study population.

The major complication related to treatment was a femoral fracture that was seen in three cases; these occurred due to the combination of postoperative weight bearing and a large cortical bone window. These three complications occurred in both medical centers and were within the first treated cases. After studying these fractures, we have learned that, despite non-weight-bearing mobilization instructions, fractures can occur due to decreased bone strength caused by the defect size. Reducing the width and, if required, increasing the length of the cortical window results in a reduced circumferential defect, which leads to an increase in bone strength. After the adjustment of this surgical step, no additional fractures occurred. Bone healing or bone defect filling after implantation of S53P4 BAG was not studied since the bone defect sizes and location were too heterogeneous, and the follow-up might be not long enough to draw conclusions from these data in this cohort study. The prolonged wound problems described in six patients with a minor complication were not directly related to local toxic effects of the S53P4 BAG, since these patients did not have any exudate formation or other local toxic signs after surgery. These patients either had a persisting fistula due to prolonged serosanguinolent leakage related to a persistent infection, or they had wound problems due to failed primary closure and the necessity for secondary closure by a plastic surgeon. In addition to the results of this study, there are no complications related to local (toxic) adverse events described in clinical and preclinical studies so far (Detsch et al., 2014; Vallittu et al., 2020; van Gestel et al., 2015).

This study has some limitations. Although the mean clinical follow-up time of 46 months is significantly longer than most comparable studies, it is still relatively short (Ferrando et al., 2017; Lindfors et al., 2010; McKee et al., 2010; Oosthuysen et al., 2020). Most cases of reinfection occur within the first 2 years after treatment, but it is known that recurrence of chronic osteomyelitis is also reported up to even 50 years after initial treatment (Korovessis et al., 1991). Another limitation of this study is the study design and the lack of a control group to compare the one-stage treatment algorithm using S53P4 BAG with the two-stage treatment algorithm using antibiotic-loaded PMMA and a bone graft. However, this was previously studied in a cost-effectiveness study, which showed good clinical and cost-effectiveness in the treatment of chronic osteomyelitis with S53P4 BAG in a one-stage fashion compared to a two-stage procedure using antibiotic-loaded PMMA beads (Geurts et al., 2019).

When comparing the clinical results to previously published results regarding treatment of chronic osteomyelitis with S53P4 BAG, we can conclude that these conform with data from previously published short-term studies. These studies followed their patients from 12 months up to 24 months, with eradication rates varying from 80 % up to 100 % (Ferrando et al., 2017; Lindfors et al., 2010; McKee et al., 2010; Oosthuysen et al., 2020). Chronic osteomyelitis was treated in a similar one-stage treatment protocol and included patients with comparable baseline characteristics (e.g., Cierny–Mader classification, location, causative pathogens, etc.). The complication rates and type of complications of this study were comparable to the larger cohort study of Lindfors et al. (2017). They also looked into possible risk factors related to the failure of treatment and found that the necessity for a soft tissue coverage by a plastic surgeon was the only major risk factor which was associated with higher reinfection rates, which is again confirmed in this study. Another potential risk factor for recurrence of infection was the combination of *pseudomonas aeruginosa* and *staphylococcus aureus* as causative pathogens, although these data are not substantiated in the present study, since this specific combination of pathogens is not seen in this study population.

There are two smaller cohort studies with minimal, 1-year follow-up results which have compared the treatment of chronic osteomyelitis with S53P4 BAG with different ceramic antibacterial bone graft substitutes (Ferrando et al., 2017; Romano et al., 2014). Results of these studies show similar eradication and complication rates, but the rates of prolonged wound leakage due to seroma might be reduced when using S53P4 BAG although this is never being studied directly. When comparing this study results to other studies solely considering different antibiotic-loaded bone graft substitutes (e.g., calcium sulfates and calcium phosphates), eradication rates remain comparable. These eradication rates varied from 85 %–96 %, in larger studies, to 100%, in smaller studies (Ferguson et al., 2014; McNally et al., 2016; Visani et al., 2018). When comparing complications, fracture and other major complication rates are similar, but the tendency for prolonged wound leakage due to seroma is also reported in these studies (Ferguson et al., 2014; Humm et al., 2014; McNally et al., 2016).

Although a one-stage treatment of chronic osteomyelitis with S53P4 BAG has comparable success and complication rates compared to other antibiotic-loaded bone graft substitutes, a major benefit of the application of S53P4 BAG is its antibacterial working mechanism. In the era of increasing antimicrobial resistance (AMR) of microbes, alternative antibacterial treatments like S53P4 BAG implantation are necessary. Also, in cases of the recurrence of infection, AMR is a challenging complication. In contrast to the recurrence of infection after treatment with S53P4 BAG and recurrence after treatment with antibiotic-loaded bone graft substitutes, using AMR might lead to treatment difficulties and less antibiotic susceptibility and/or antibiotic options. In addition to this major advantage, a one-stage treatment algorithm with S53P4 bioactive glass also gives a financial/health economics advantages, since the duration hospital of stay and the concomitant treatment related costs are significantly reduced in comparison to a two-stage treatment with antibiotic-loaded PMMA beads (Geurts et al., 2019).

## 5 Conclusion

The introduction of S53P4 bioactive glass enabled the treatment of chronic osteomyelitis in a one-stage fashion. This study presents good results in the eradication of infection and shows low complication rates in a large study population after an average 46-month clinical follow-up period. A major risk factor for the failure of treatment is a poor soft tissue status necessitating a full thickness skin graft or vascularized (free) muscle flap. Despite these satisfying data, future research should be performed in order to decrease recurrence of infection rates in the short-term and long-term and also by addressing major importance soft tissue problems within these chronic multimodal infections. The findings in this prospective study further substantiate previously published short-term data on the one-stage treatment of chronic osteomyelitis using S53P4 bioactive glass.

## Data Availability

Data are not available since it is specific patient record data only available in the hospital. But the data sets analyzed during the current study are available from the corresponding author on reasonable request.
